# Isolation of β-1,3-Glucanase-Producing Microorganisms from *Poria cocos* Cultivation Soil via Molecular Biology

**DOI:** 10.3390/molecules23071555

**Published:** 2018-06-27

**Authors:** Qiulan Wu, Xin Dou, Qi Wang, Zhengbing Guan, Yujie Cai, Xiangru Liao

**Affiliations:** The Key Laboratory of Industrial Biotechnology, Ministry of Education, School of Biotechnology, Jiangnan University, 1800 Lihu Road, Wuxi 214122, China; 15861667099@163.com (Q.W.); xin_dou@163.com (X.D.); zhiwen_wei@126.com (Q.W.); 15061886046@163.com (Z.G.); xjj491202524@126.com (Y.C.)

**Keywords:** β-1,3-glucanase, gene cloned and expressed, high-throughput sequencing, actinomycetes

## Abstract

β-1,3-Glucanase is considered as a useful enzymatic tool for β-1,3-glucan degradation to produce (1→3)-linked β-glucan oligosaccharides with pharmacological activity properties. To validly isolate β-1,3-glucanase-producing microorganisms, the soil of *Wolfiporia extensa,* considered an environment rich in β-1,3-glucan-degrading microorganisms, was subjected to high throughput sequencing. The results demonstrated that the genera *Streptomyces* (1.90%) and *Arthrobacter* (0.78%) belonging to the order Actinomycetales (8.64%) in the phylum Actinobacteria (18.64%) were observed in soil for *P. cocos* cultivation (FTL_1_). Actinomycetes were considered as the candidates for isolation of glucan-degrading microorganisms. Out of 58 isolates, only 11 exhibited β-1,3-glucan-degrading activity. The isolate SYBCQL belonging to the genus *Kitasatospora* with β-1,3-glucan-degrading activity was found and reported for the first time and the isolate SYBC17 displayed the highest yield (1.02 U/mg) among the isolates. To check the β-1,3-glucanase contribution to β-1,3-glucan-degrading activity, two genes, 17-W and 17-Q, encoding β-1,3-glucanase in SYBC17 and one gene QLK1 in SYBCQL were cloned and expressed for verification at the molecular level. Our findings collectively showed that the isolates able to secrete β-1,3-glucanase could be obtained with the assistance of high-throughput sequencing and genes expression analysis. These methods provided technical support for isolating β-1,3-glucanase-producing microorganisms.

## 1. Introduction

The glycoside hydrolase β-1,3-glucanase, extensively distributed among plants, fungi, and bacteria, acts on 1,3-β-glucosidic bonds of structural β-1,3-glucans to hydrolyze or transfer glycosides [1,2]. Based on the hydrolysis position, β-1,3-glucanases are divided into endo-type (E.C. 3.2.1.39) and exo-type (E.C 3.2.1.58) categories. β-1,3-Glucans are the main cell wall component.β-1,3-Glucanase is able to hydrolyze these β-1,3-glucans, as a defense against fungal pathogens in plants, as well as to suppress fungal growth in fermentation technology [3,4]. Moreover, β-1,3-glucans can be degraded into (1→3)-linked β-d-glucan oligosaccharides with immunomodulating and antitumor properties by β-1,3-glucanase [5,6]. The exo-β-1,3-glucanase gene encodes an immune-dominant protein as a cell wall remodeler, which is a candidate for immunotherapy and can be used in diagnostic tests [7].

In bacteria, numerous recombinant and wild enzymes have been characterized from different sources, e.g., *Streptomyces* [8], *Arthrobacter* sp. [9,10], *Cellulosimicrobium cellulans* [11], *Nocardiopsis* sp. [12], *Paenibacillus* sp. [13], *Thermotoga neapolitana* [14], *Bacillus circulan* [15]. However, no reports have been made on β-1,3-glucanase selected from the genus *Kitasatospora*. β-1,3-Glucanase originating from bacteria are mainly classified into glycosyl hydrolase family 16, according to the putative amino acid sequences and secondary structures. The methods of isolating bacteria able to secrete β-1,3-glucanase were based on the lysing of cell walls of higher fungi or degrading polysaccharides [16,17]. Glucanase-producing microorganisms belonging to pathogenic bacteria were often selected in these way, and other isolates able to secrete β-1,3-glucanase were covered or their growth was inhibited. Although the methods of isolating strains with glucanase activity have been described, detailed data on the diversity of glucanase-producing microorganisms in any specific environment, which would be beneficial to screen the isolates able to secrete β-1,3-glucanase, are unavailable. 

*P. cocos*, a fungus consisting of 90% β-glucan and 10% terpenes by dry weight, grows around the roots of pine trees. Linear 1,3-β-d-glucans with some glucose branches as the main component were extracted from *P. cocos* sclerotium [18]. Plant growth influences the diversity of rhizosphere microorganisms [19]. The soil for *P. cocos* cultivation is more likely to harbor diverse populations of bacteria with β-1,3-glucanase for polysaccharides hydrolysis.

Recent advancements have been made in high-throughput sequencing, which can provide sequencing depths to cover various bacterial ecosystems, e.g., soil, sediment, animal, insect gut, marine or freshwater [20,21,22,23,24]. The combination of high-throughput sequencing and culture-dependent techniques was conducted to characterize the microbial communities colonizing ancient Buddhist statues [25] as well as to construct a novel bioassay for *N*-acyl homoserine lactone-degrading microorganisms [26].

The aims of this study were to: (1) analyze the diversity and structure of microbial communities by using the method of high-throughput sequencing, (2) isolate and identify glucan-degrading actinomycetes, (3) determinate β-1,3-glucan-degrading activity, and (4) clone and express the genes encoding β-1,3-glucanase.

## 2. Results and Discussion

### 2.1. Bacterial Community Analysis

By the Miseq sequencing analysis of soil samples, 103,094 raw reads were generated. After trimming, denoising, and filtering chimeras, 102,005 effective sequences remained with the average length of 450 bp. Subsequently, the remaining sequences were clustered by 3% dissimilarity, and 18,446 bacterial operational taxonomic units (OTUs) were identified. The numbers of OTUs, Good’s coverage, Chao1 parameters, and Shannon indexes are collected in Table 1. The OTUs obtained from soil samples are displayed using rarefaction curves (Figure S1).

**Figure 1:**
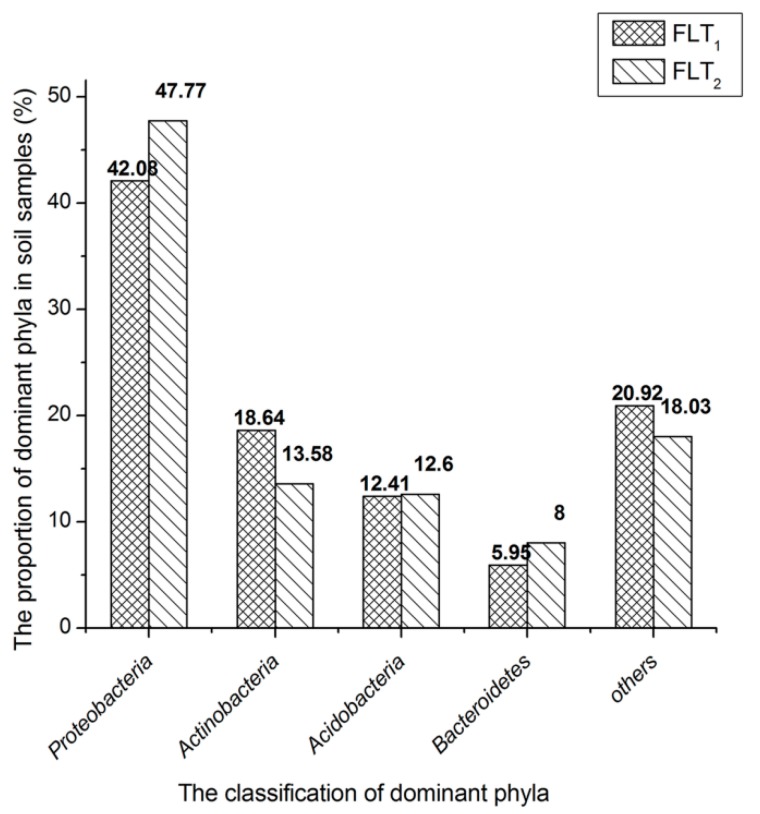
Phylum-level microbial communities in soil samples. The taxa represented account for >5% abundance in at least one sample. Other phyla represent the taxa with their maximum abundance of <5% in any sample.

The classification results at the taxa phylum level are depicted in Figure 1. Proteobacteria (42.08%), Actinobacteria (18.64%), Acidobacteria (12.41%) and Bacteroidetes (5.95%) are considered as the dominant phyla as they contained over 5% of high-quality sequences in FLT_1_. The high-quality sequences not less than 1% were also classified into other subdominant phyla (Table S1). Eleven of abovementioned groups accounted for 96.13%. The remaining 16 phyla, in which effective sequences occurred at <1% abundance of the high-quality sequences, were defined as rare phyla in FLT_1_. Euryarchaeota and Thaumarchaeota belonging to the archaea domain accounted for 0.04% and 0.01% of the total high-quality sequences among FLT_1_, respectively (Table S1). The dominant phyla of FLT_1_ and FLT_2_ were compared. The composition of dominant phyla in FLT_2_ was similar to those of FLT_1_. The phylum Actinobacteria in FLT_1_ was over 5.06% of high-quality sequences those in FLT_2_. The abundances of other dominant phyla in FLT_2_ were greater than those in FLT_1_. Root exudates containing various primary and secondary plant metabolites have distinct influences on insect herbivores, nematodes, and microbes underground, in addition to deterring competing plants [27]. Thus, the microbial communities in FTL_1_ and FTL_2_ might illustrate the relationship of the abundances of dominant phyla with its use of the main metabolites produced by *P. cocos.* The abundance of Actinobacteria is more likely to relate with β-1,3-glucan produced by *P. cocos*. It is reasonable to consider that bacteria belonging to the phylum Actinobacteria probably have ability to secrete β-1,3-glucanase for β-1,3-glucan degradation. Certainly, it cannot be excluded that other dominant phyla can found microorganisms with β-1,3-glucanase activity.

**Figure 2:**
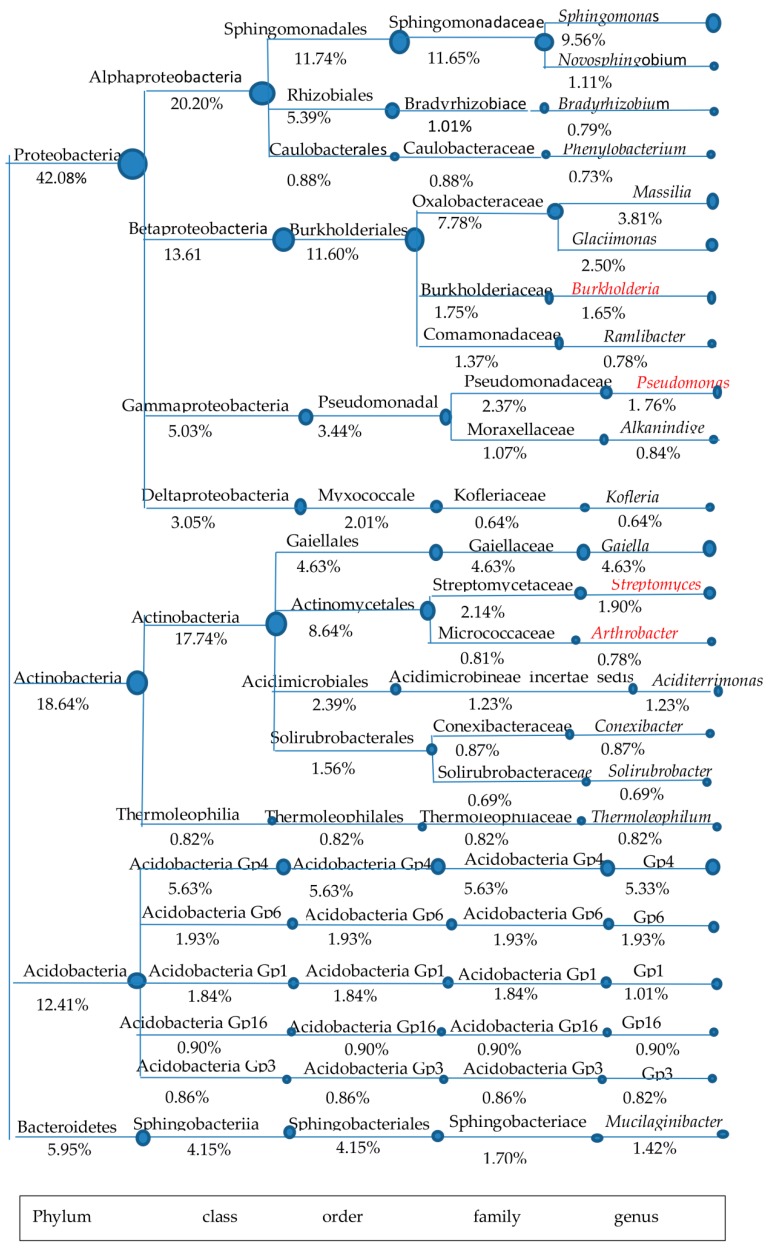
A tree was composed of classification systems within dominant phyla at the genus level in FTL_1_. The abundances of taxa genera are over the top 30. The genera with bacterial β-1,3-glucanase are marked in red.

The soil for *P. cocos* cultivation (FLT_1_) was used as the candidate sample for isolating β-1,3-glucanase-producing microorganisms. To understand the diversity and structure of β-1,3-glucanase-producing microorganisms in FLT_1_, a combination of literature research and data analysis among dominant phyla was conducted in detail (Figure 2). Although Proteobacteria is the main dominant phylum that exists in FLT_1_, few reports refer to β-1,3-glucanase-producing microorganisms belonging to the phylum Proteobacteria from FLT_1_. Indeed, detailed data about microorganisms able to secrete glycoside hydrolases are available for this phylum, e.g., *Sphingomonas* [28,29], Sphingomonadaceae [30], *Burkholderia* [31], *Pseudomonas* [32].

Only the glycoside hydrolases selected from the genera *Pseudomonas* and *Burkholderia* that act on β-1,3-glucan have been exhaustively reviewed. The genus *Burkholderia* is worthless to isolate for its pathogenicity. Rare organisms able to degrade β-1,3-glucan were observed in the phyla Acidobacteria and Bacteroidetes from FLT_1_, except a characterized β-glucosidase from Mucilaginibacter sp. Strain QM49 [33]. The genera *Arthrobacter* and *Streptomyces*, accounting for 0.78% and 1.90% of the total high-quality sequences, were observed in the phylum Actinobacteria. It was noteworthy that both of the genera belong to the order Actinomycetales, which have been extensively reported as a source of β-1,3-glucanase [34,35,36]. FLT_1_ widely harbored the order Actinomycetales (8.64%) when it was compared with others especially the genus *Pseudomonas* (1.76%) belonging to the order Pseudomonadales (3.44%). Moreover, the remarkable presence of Actinobacteria was found in FLT_1_ but few that of microorganisms were observed in FTL_2_. A member of the order Actinomycetales is often called an actinomycete. It is well known that actinomycetes have unrivalled capacity to produce over two-thirds of natural antifungal metabolites [37]. Actinomycetes of the genus *Streptomyces* is well known as the largest genus of Actinobacteria, with properties of biological control. Overall, FLT_1_ probably harbored a number of actinomycetes able to secrete β-1,3-glucanase. Actinomycetes can be classified into probiotics. Thus, the soil for *P. cocos* cultivation was used as the candidate sample for isolating β-1,3-glucanase-producing actinomycetes.

### 2.2. Identification of Glucan-Degrading Microorganisms

**Figure 3:**
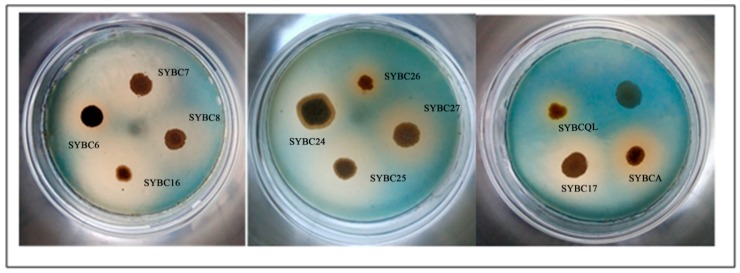
Glucan-degrading actinomycetes formed a clear halo around the colony.

Colonies of actinomycetes were visible after the dilution of soil cultured on yeast casamino acids extract and dextrose agar (YCED). The isolates with β-1,3-glucan-degrading activity were screened among preferred actinomycetes. Out of 58 actinomycetes, only 11 among formed a clear halo around the colony after inoculation, indicating that they were able to degrade glucan (Figure 3). A positively relation can be found between the size of clear halo and the enzyme activity. The size of clear halo in SYBC26 and SYBCQL were obviously smaller than others. Thus both of them are weak to degrade β-1,3-glucan.

**Figure 4:**
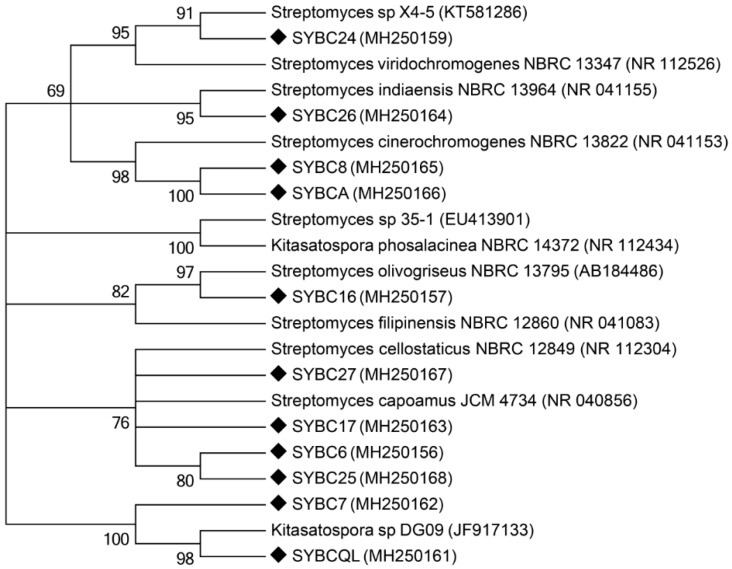
Phylogenetic analysis of 16S rRNA gene sequences of the isolates from FTL_1_. A neighbor-joining tree was obtained from a BLAST search of 16S rRNA gene sequences of the isolates for phylogenetic inference. The bootstrap values presented at corresponding branches were evaluated using 1000 replicates.

Ten isolates were identified to the genus *Streptomyces*, with the exception of SYBCQL belonging to the genus *Kitasatospora*, via 16S rRNA gene analysis. Each of the 16S rRNA gene sequences from isolates were aligned and submitted to the GenBank database, and all the sequences showed a high identity match (99%) to sequences obtained from the GenBank database (Table 2). Four isolates were closer to *S. cellostaticus* and *S. capoamus*. Two isolates showed high similarities to *S. cinerochromogenes* and *S. coelescens*. Three isolates were homologous to *S. indiaensis*, *S. viridochromogenes*, and *K. phosalacinea,* respectively, while another was homologous to *S. olivogriseus* and *S. filipinensis*. Phylogenetic analysis verified the taxonomic affiliations searched by BLAST alignment (Figure 4). The genus *Streptomyces* as a main member of actinomycetes was abundant in FLT_1_ based on high-throughput sequencing. 

The results of identification were in agreement with the analysis of microbial communities at the genus level in FTL_1_**.** The genus *Kitasatospora*, is homologous to the genus *Streptomyces*, belonging to the order Actinomycetales among the phylum Actinobacteria [38]. Thus, SYBCQL able to degrade β-1,3-glucan was isolated from FLT_1_ under the same conditions.

### 2.3. Enzyme Activity Assay

The isolates that formed a clear halo around the colony (Figure 3) were determined to have the ability to degrade glucan in an exhausted culturing medium (Table 3). The isolates able to degrade β-1,3-glucan were feeble, as compared with that of *Streptomyces rutgersensis* [39] and *Streptomyces torulosus* PCPOK-0324 [16]. SYBC17 showed the highest yield of glucan-degrading activity (1.02 U/mg) among all isolates obtained from actinomycetes. Although the specific activity of SYBCQL was lower than that of others, the genus *Kitasatospora* with β-1,3-glucan-degrading activity was found and reported for the first time. In general, β-glucosidases participate in β-1,3-glucan degradation along with β-1,3-glucanases. Thus SYBCQL and SYBC17 with β-1,3-glucanases activity for β-1,3-glucan degradation were further verify at the molecular level.

### 2.4. Gene Clone and Analysis

One gene encoding β-1,3-glucanase was amplified from the genomic DNA of SYBCQL and named *QLK1,* encoding the deduced protein QLK1. Based on the genomic DNA of SYBC17, two β-1,3-glucanase genes were found and named *17-W*, and *17-Q*. Both correspond to the deduced proteins 17-W and 17-Q. PCR products were checked by 1% agarose gel electrophoresis and sequenced after TA cloning. 

**Figure 5:**
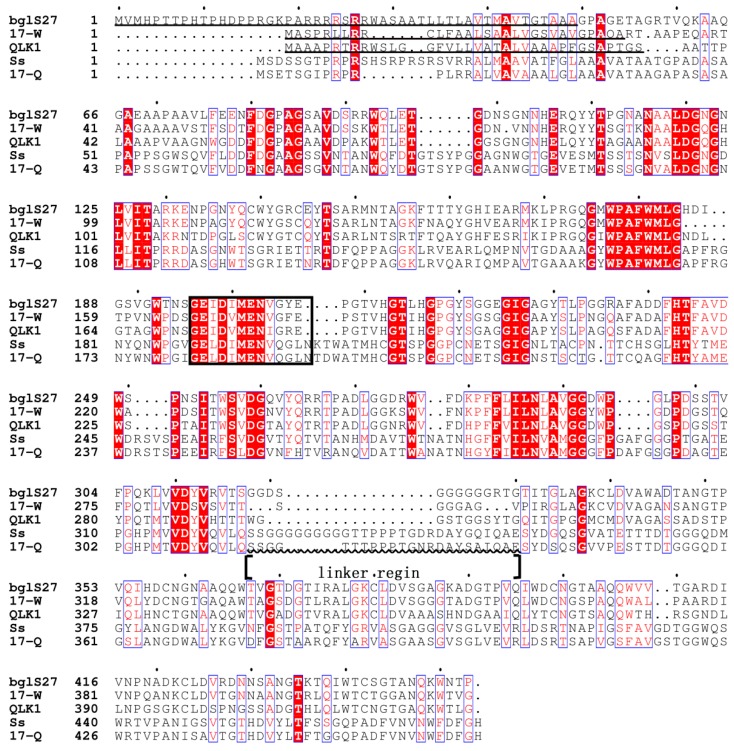
Alignment of SYBCQL and SYBC17 enzymes with other β-1,3-glucanases of GHF16. 17-W and 17-Q, SYBC17; QLK1, SYBCQL; bgl27, *Streptomyces* sp. S27; Ss, *Streptomyces sioyaensis*. Sequences were taken from the following accession numbers: *Streptomyces* sp. S27 endo-β-1,3-glucanase (FJ887899); *S. sioyaensis* endo-β-1,3-glucanase (AF217415). The deduced amino acid signal peptides are underlined. The putative catalytic motif residues are boxed in black. The deduced location of linker regions are indicated by a wavy line.

The residues 1–37 of QLK1 and the residues 1–30 of 17-W was identified as *N*-terminal signal peptides, according to SignaIP analysis. The mature protein QLK1 consisted of 391 residues with a deduced molecular mass of 40.4 kDa. The mature protein 17-W contained 389 residues and its deduced molecular mass was the same as QLK1. Meanwhile, 17-Q without an *N*-terminus leader sequence encoded a mature protein with a deduced molecular mass of 48.1 kDa (Figure 5). Each of the putative amino acid sequences has a catalytic domain similar to GH 16, based on align the protein sequences from GenBank database. QLK1 and 17-W, with a potential carbohydrate-binding domain (CBM), similarly belong to the regions of CBM 13 from *Streptomyces* at the C-terminus sequence. The functional domains of QLK1 and 17-W were found to be similar to β-1,3-glucanase from *Streptomyces* sp. S27 [35]. A glycine-rich region was observed between the functional domains of GH 16 and CBM 13 in QLK1 and 17-W. The region was also found in the linker structure in β-1,3-glucanase from *Streptomyces* sp. S27 [35] and *S. sioyaensis* [8]. The C-terminus domain of 17-Q was grouped into CBM family 6, found in several xylanases, rather than CBM family 13. The functional domains of 17-Q was similar to β-1,3-glucanase from *S. sioyaensis* [8]. 

**Figure 6:**
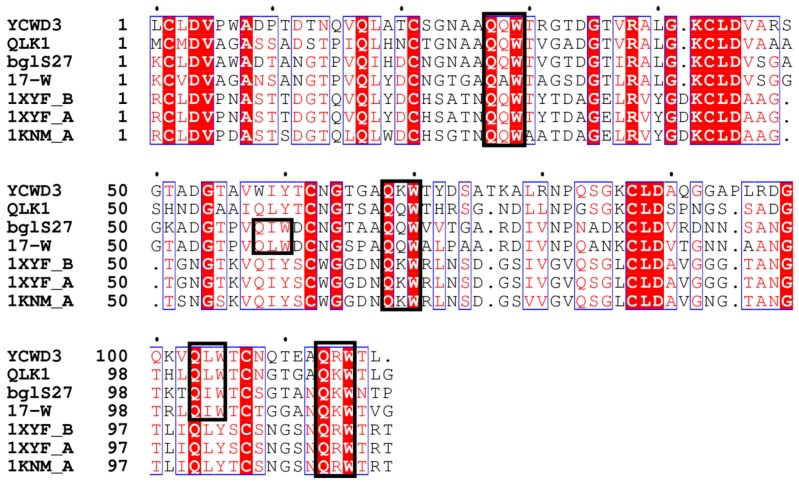
Partial alignment of the C-terminal carbohydrate-binding domain (CBM) of SYBCQL and SYBC17 enzymes with several CBMs found in xylanases and endo-β-1,3-glucanases. QLK1 β-1,3-glucanase, SYBCQL; 17-W β-1,3-glucanase, SYBC17; bgl27 endo-β-1,3-d-glucanase, *Streptomyces* sp. S27 (FJ887899); YCWD3 β-1,3-glucanase, *Arthrobacter* sp. YCWD3 (D23668); 1KNM_A xylanase 10A, *Streptomyces lividans* (M64551); 1XYF_A Endo-β-1,4-Xylanase Chain A, *Streptomyces Olivaceoviridis* (PDB entry: 1XYF_A); 1XYF_B Endo-β-1,4-Xylanase Chain B, *Streptomyces Olivaceoviridis* (PDB entry: 1XYF_B). The Gln-X-Trp repeats are boxed in black.

**Figure 7:**
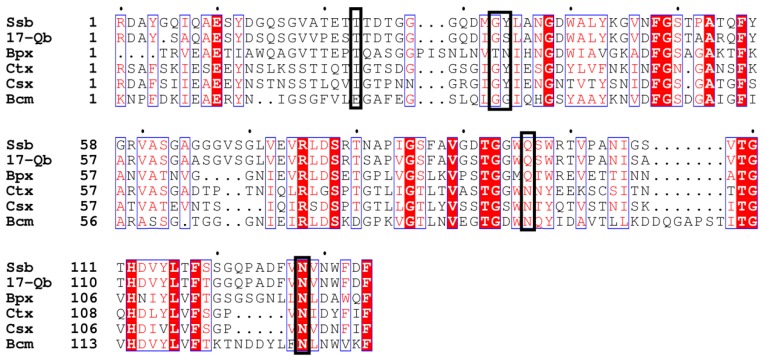
Partial alignment of the C-terminal CBM of SYBC17 enzyme with several CBMs found in xylanases and endo-β-1,3-glucanases. 17-Q β-1,3-glucanase, SYBC17; *Ssb* endo-β-1,3-glucanase, *Streptomyces sioyaensis* (AF217415); Ctx xylanase A, *Clostridium thermocellum* F1/YS (AF04776); Csx xylanase A, *Clostridium stercorarium* F-9 (D13325); Bpx xylanase D, *Bacillus polymyxa* (X57094); Bcm α-1,6-mannanase, *Bacillus circulans* TN31(AB024331). The ligand binding sites are boxed in black.

The CBMs of QLK1 and 17-W exhibited a structure like that of the ricin B-chain classified in CBM 13 members. The ricin B lectin domain is composed of three homologous regions as the QXW (Gln-X-Trp) repeats (Figure 6) [40]. There is a hypothesis that Gln works in substrate binding and Trp help to form the hydro phobic core [41]. The CBM of 17-Q is homogeneous to the CBMs belonging to family 6. Generally, members of CBM family 6 bind to xylan by connecting with the xylanase domain. For instance, the CBM of xylanase A from *Clostridium stercorarium* has been suggested to bind xylan and act as an important role in xylan hydrolysis [42]. Family 6 CBMs containing multiple distinct ligand binding sites present a unique ligand binding surface to recognize the non-reducing end of β-1,3-linked-glucans [43]. The CBM of *S. sioyaensis* β-1,3-glucanase is probably considered as an extra ordinary CBM classified into family 6, based on its binding preference, especially due to its unwilling binding to xylan (Figure 7) [8]. The ligand binding sites of 17-Q are similar to those found in the CBM of *S. sioyaensis* β-1,3-glucanase and probably have the same binding preference.

A highly consensus catalytic center for the hydrolysis of glycosidic bonds has been observed in GH family 16 [44,45]. A Met residue was observed in the catalytic motif of endo-β-1,3-glucanases but not in endo-β-1,3-1,4-glucanases [11]. As shown in Figure 5, a specific consensus motif with putative catalytic residues is found among these β-1,3-glucanases. BglF is completely inactive when the mutants of the deduced catalytic residues Glu123Gln and Glu128Gln are created [12]. Thus, the putative catalytic residues are crucial among these hydrolases. Besides, Glu128 protonates the glycosidic oxygen of the scissile bond by acting as a general acid [44].

**Figure 8:**
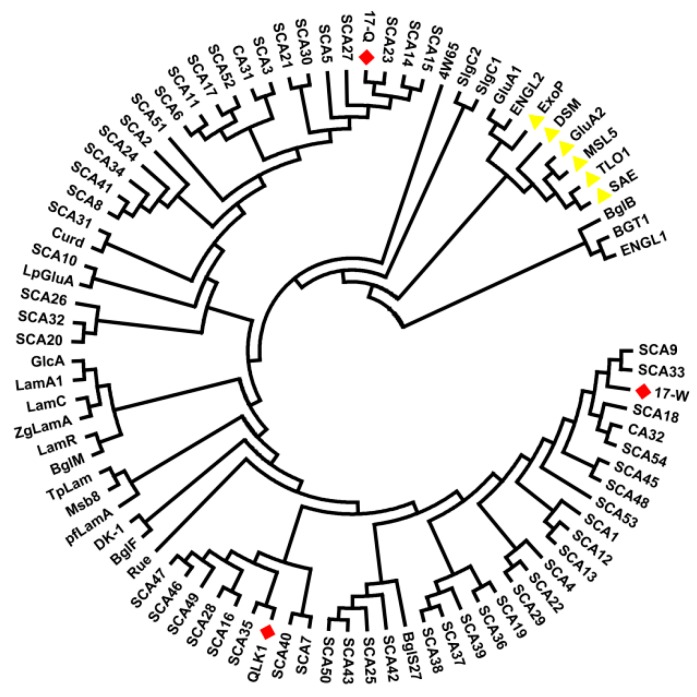
Phylogenetic tree of some amino acid sequences of β-1,3-glucanase. The deduced amino acid sequences of β-1,3-glucanase from SYBCQL and SYBC17 are marked in red. Glycoside hydrolases belonging to the exo-type clade are marked in yellow and others are divided into endo-types.

The open reading frames (ORF) were aligned by using the online tool BLAST. QLK1 was 64% identical to the putative secreted hydrolase from *S. coelicolor* A3(2). Furthermore, QLK1 was homogeneous to β-1,3-glucanase from *Nocardiopsis* sp. strain F96 (63%) and *Streptomyces* sp. S27 (64%). 17-W was 84% identical to the putative secreted hydrolase from *S. coelicolor* A3(2) and showed identity with β-1,3-glucanase from *Streptomyces* sp. S27 (69%) and *Nocardiopsis* sp. strain F96 (53%). 17-Q showed identity with the putative secreted glucosidase from *S. coelicolor* A3(2) (80%) and endo-β-1,3-glucanase from *S. sioyaensis* (79%) and was 57% identical to *Arthrobacter* sp.NHB-10. The results indicated that QLK1 and 17-W were closer to the putative secreted hydrolase from *S. coelicolor* A3(2) and was significantly different from 17-Q. While 17-Q was similar to the putative secreted glucosidase from *S. coelicolor* A3(2) and endo-β-1,3-glucanase from *S. sioyaensis* (Figure 8 and Table 4). Interestingly, high homology was observed between the ORFS and those of never identified β-1,3-glucanase according to phylogenetic analysis.

### 2.5. Expression and Purification of Recombinant Enzymes 

**Figure 9:**
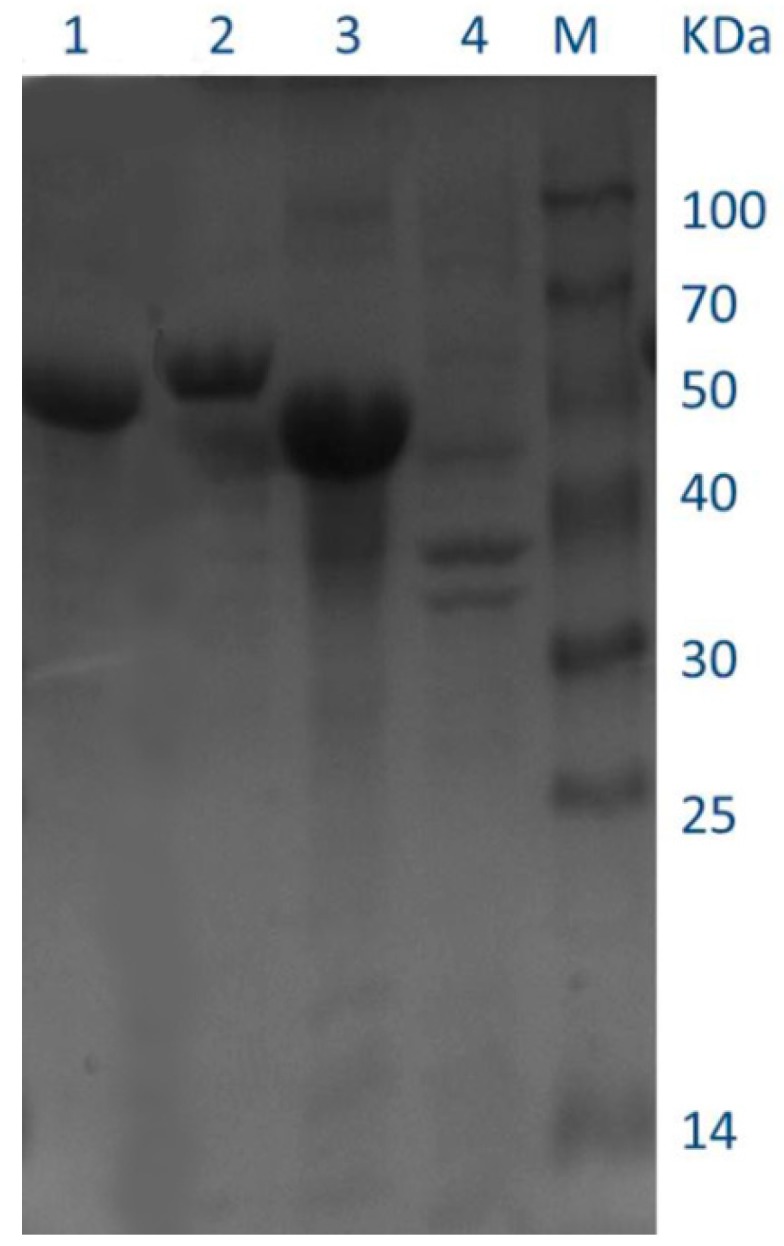
SDS-PAGE analysis of the purified recombinant enzymes overexpressed in *E. coli* BL21 (DE3). Lanes: M, molecular mass markers; 1, purified recombinant QLK1; 2, purified recombinant 17-Q; 3, purified recombinant 17-W; 4, culture supernatant of the induced transformant harboring empty pcold II.

Recombinant enzymes were expressed with IPTG (isopropyl-β-d-1-thiogalacto-pyranoside) induction in *E. coli* BL21 (DE3) cells. Crude extracts in soluble form were purified using Ni^+^ affinity chromatography and desalting chromatography was used to remove the excess salt. SDS-PAGE analysis confirmed that QLK1, 17-W and 17-Q were overexpressed successfully, with high purity of the enzymes. The presence of molecular masses was close to the theoretical masses according to the deduced amino acid sequences of the enzymes (Figure 9). The purified recombinants QLK1, 17-W and 17-Q had specific activities of 65.82 U/mg, 132.90 U/mg, and 14.70 U/mg, respectively (Table 5). 17-Q showed the highest yield among all purified recombinant enzymes. All the recombinant enzymes displayed a several times higher level of β-1,3-glucanase activity than wild-type. These results suggested that SYBCQL and SYBC17 with β-1,3-glucanases activity were successfully confirmed in molecular level.

## 3. Materials and Methods

### 3.1. Materials

SYBCQL and SYBC17 were used as genetic DNA sources. *E. coli* DH5α and *E. coli* BL21 (DE3) were purchased from TaKaRa (Dalian, China) and used as hosts for genes cloning and expression. The plasmid pUC19 and pCold II vector were bought from TaKaRa and used for constructing recombinant plasmid. Luria-Bertani (LB) medium with 50 μg/mL ampicillin was used in recombinant plasmid amplification. The genomic DNA extraction kit, LA Taq DNA polymerase with GC buffer, PCR clean-up kit and other DNA-modifying enzymes were bought from TaKaRa. Laminarin with an average BR of 98% was purchased from Shanghai Yuanye Bio-Technology Company (Shanghai, China). The powder of fruiting bodies of the *Basidiomycete P. cocos* was provided by Johncan International Company (Hangzhou, China). High-throughput sequencing was performed by Shanghai Shenggong Company (Shanghai, China). Other chemicals were all of analytical grade and commercially available.

### 3.2. High-Throughput Sequencing

To find the candidate isolates in FLT_1_, for screening β-1,3-glucanase-producing microorganisms, the microbial communities in the soil for *P. cocos* cultivation (FLT_1_) and bulk soil (FLT_2_) were investigated and compared. 

Soil sampling was carried out in March 2017. The sphagnum and duff layers of the sampling area were removed, and *P. cocos* were found around the roots of pine trees using a soil knife. FLT_1_ was collected from the soil around *P. cocos* growing in a township (28°35′ N, 185°95′ E), Liu’an City, Anhui Province, China. The bulk soil sample (FLT_2_) was gathered approximately 2 m away from FLT_1_ and just under the root zone of any grasses growing on the surface (pH 7.0). The soil of the 10 cm depth layer was collected using an auger with a diameter. To remove stones and roots, both of the soil samples were timely sieved (2 mm mesh) in the field. The treated samples were then kept under a low temperature maintained by ice until molecular analysis. 

High-throughput sequencing in molecular analysis and data processing were conducted as described previously [46]. After DNA extraction, PCR amplification, and pyrosequencing, the MiSeq-generated raw sequences were submitted to the DDBJ database (accession number: DRA006753). The raw MiSeq-generated sequences were further processed using the soft-ware Prinseq (PRINSEQ-lite 0.19.5) [47] and the software package of Mothur1.30 with “*pre.cluster*” command [48]. The available sequences were clustered into operational taxonomic units (OTUs) and the thresthod value of sequences similarity was set at 0.97. Based on the results of OTU clustering, the most abundant sequence as the representative sequence of OTU was acquired and subjected to various types of analysis.

Taxonomic assignment was accomplished by the Ribosomal Database Project (RDP) Classifier according to Bergey’s taxonomy [49]. A bootstrap cutoff of 80% was used to assign the obtained sequences to each taxonomy levels. The evolutionary relationships and abundance of the dominant phyla in FLT_1_ at the genus level were visualized using the ete3 (Environment for Tree Exploration) package in python. 

### 3.3. Isolation and Identification of Glucan-Degrading Microorganisms

Actinomycetes were screened from FLT_1_ by serial dilution and spread-plate techniques [50]. FLT_1_ (5 g) was mixed with 100 mL sterile distilled water and diluted to 10^−5^. One hundred microliter of the different dilutions were grown on the plates containing 0.03% yeast extract, 0.03% casamino acid, 0.03% d-glucose, 0.05% K_2_HPO_4_, and 1.8% agar (*w/v*) in triplicates, respectively. Cyclohexamide (100 μg/mL) was added to resist fungal contamination after autoclaving. The plates were incubated at 28 °C for 1–2 weeks. Typical actinomycetes colonies were picked out according to morphological characteristics as well as microscopic examination. The morphologically distinct colonies were then purified on the original media at 28 °C for one week, stored at 4 °C. Furthermore, glucan-degrading actinomycetes were inoculated on agar plates containing the powder of fruiting bodies of *Basidiomycete P. cocos* (0.5%, *w*/*v*) and aniline blue (0.005%, *w*/*v*), and then formed a clear halo around the colony [51]. 

To identify the unknown isolates, the selected isolates were incubated in 3 mL ISP-2 medium under rotary shaking at 30 °C for 48 h. A volume of 1.5 mL culture was centrifuged at 8000× *g* for 1 min. The pellet was then washed once with distilled water and used to extract genomic DNA. The genomes of each isolate were extracted following by the operating instruction of genomic DNA extraction kit (www.tiandz.com). 16S rRNA gene identification of glucanase-producing bacteria were amplified by using the universal primers 27F (5′-AGAGTTTGATCCTGGCTCAG-3′) and 1492R (5′-GGTTACCTTGTTACGACTT-3′) and sent to Shanghai Shengong (Shanghai, China) for sequencing. A homology search of the closest phylogenetic neighbors was conducted using the online tool BLAST.

### 3.4. Determination of the Enzyme Activity 

The isolates were inoculated aerobically with rotary shaking at 200 rpm and 30 °C for 72 h in a medium (per liter) containing laminarin 5 g, K_2_HPO_4_ 1 g, NaNO_3_ 3 g, KCl 0.5 g, MgSO_4_·7H_2_O 0.5 g, and FeSO_4_·7H_2_O 0.5 g. The cultures were centrifuged at 10,000× *g* at 4 °C and the culture filtrates were harvested for activity assay. The standard activity assay for β-1,3-glucan degradation was obtained by measuring the formation of reducing sugar using a colorimetric method [16]. Culture filtrates of the strains (500 μL) were mixed with 500 μL of 0.5% (*w*/*v*) laminarin in 100 mM sodium acetate buffer (pH 5.5). The reaction was conducted at 50 °C for 60 min and terminated by heating for 5 min at 100 °C. Then 2 mL of 1% dinitrosalicylate (DNS) was added into the reaction solution and the mixture was boiled for 10 min. The mixture was placed in an ice bath and then measured at 540 nm using the spectrophotometer. According to the standard assay conditions, one unit (U) of the activity was defined as the amount of enzymes that can liberate 1 μmol of glucose in one minute. All experiments were set to repeat, with triplicates of each treatment. Protein concentrations were measured by the method of Bradford [52] using bovine serum albumin as a standard.

### 3.5. Cloning and Expression of β-1,3-Glucanase Genes

A genome analysis from the NCBI database was conducted, with the genomic DNA of *Kitasatospora setae* KM-6054 and *Streptomyces griseochromogenes* ATCC 14511 employed as the templates and synthetic primers (Table 6). The genome of SYBCQL and SYBC17 has been extracted by genomic DNA extraction kit. The plasmid pCold II DNA and the vector pUC19 DNA was isolated using a plasmid miniprep kit. Then the coding sequences were amplified by the polymerase chain reaction (PCR) using LA Taq DNA polymerase with GC buffer, and sequenced after TA cloning. The nucleotide sequences were deposited in the GenBank database (accession number: 17-W, MH190407; 17-Q, MH190408; QLK1, MH190409.). Subsequently PCR products were ligated with the pcold II vector after both were digested with *Hind* III and *Xba* I. The recombinant plasmid was then transformed into *E. coli* BL21 (DE3) competent cells.

Transformants containing the recombinant enzymes were picked from the single colony and inoculated overnight at 37 °C in ampicillin-supplemented LB. Moreover, the overnight cultured transformants (1 mL) were transferred into 50 mL of fresh LB medium with the addition of 100 μg/mL ampicillin and grown at 37 °C to a cell density of 0.6~0.8. To induce the expression of the recombinant enzymes, IPTG was then added to a final concentration of 0.4 mM and the cultivation continued for 24 h at 15 °C. Cells were harvested by centrifugation at 4 °C and 8000× *g* for 10 min, and resuspended in 100 mM sodium acetate buffer at pH 5.5. Cells were lysed by sonication for 15 min on ice, and cell supernatants was collected by centrifugation (8000× *g*, 10 min at 4 °C) for further purification.

### 3.6. Purification of the β-1,3-Glucanases

To purify the recombinant proteins with six histidine residues, an AKTA Avant system at 6 °C was used (GE Healthcare, Uppsala, Sweden), followed by desalting with a HisTrap TM column (GE Healthcare, Uppsala, Sweden). The cell supernatant (crude enzyme) was applied to a HisTrap HP column (GE Healthcare) equilibrated with binding buffer A (100 mM sodium acetate, 5 mM imidazole, 500 mM NaCl, pH 5.5), and was eluted with buffer B (100 mM sodium acetate, 500 mmol/L imidazole, and 500 mmol/L NaCl, pH 5.5) using an imidazole step gradient of 0% to 100% buffer B. The collected fractions with β-1,3-glucanase activity were assayed using the former described method. The purified proteins were loaded on 12% sodium dodecyl sulfate polyacrylamide gel electrophoresis (SDS-PAGE). The concentration of purified proteins was tested by the method described above.

### 3.7. Bioinformatics Analysis

In the NCBI database (https://www.ncbi.nlm.nih.gov/genome), a homology analysis was performed using the online tool BLAST (https://blast.ncbi.nlm.nih.gov/Blast.cgi). DNA and protein sequence alignments were performed using the blastn and blastp programs in the NCBI database, respectively. The *N*-terminus signal peptide was forecasted by the SignaIP 3.0 server (http://www.cbs.dtu.dk/ services/SignaIP/). The multiple sequence alignment was performed based on ClustalW program (http://www.ebi.ac.uk/clustalW/) and embellished by ESPript (http://espript.ibcp.fr/ESPript/cgi-bin/ESPript.cgi). Phylogenetic analysis was conducted by Molecular Evolutionary Genetics Analysis (MEGA) 5.0 with neighbor-joining method at a bootstrap of 1000.

## 4. Conclusions

In the present paper, actinomycetes with the ability to degrade β-1,3-glucan were isolated using high-throughput sequencing combined with culture-dependent techniques. Both strains SYBCQL and SYBC17 able to secrete β-1,3-glucanase for β-1,3-glucan degradation were verified at the molecular level. It was suggested that these methods could be applied to effectively isolate β-1,3-glucanase-producing microorganisms, which is useful for the screening of other metabolite-producing microorganisms from specific environment. 

## Figures and Tables

**Table 1:** Bacterial richness indices of soil samples.

Samples	Number of Effective Sequences	Number of OTUs	Coverage (%)	Chao1	Shannon Index
FLT1	44,870	7737	88.51	25,534.52	6.95
FLT2	57,135	10,709	88.56	34,009.99	7.52

**Table 2:** BLAST analysis of glucan-degrading microorganisms isolated from FLT_1_.

Isolate Name	16S rRNA Gene Sequence Lengh (bp)	Best BLAST Hit(s)	Accession Number	Identity (%)
SYBCQL	1345	*Kitasatospora phosalacinea* JKCM-G-8A *Kitasatospora phosalacinea* NBRC 14372 ^a^	LC010672.1 NR_112434.1	99 99
SYBCA	1396	*Streptomyces cinerochromogenes* MC10130 *Streptomyces cinerochromogenes* NBRC 13822 ^a^ *Streptomyces coelescens* CSSP420 *Streptomyces cinerochromogenes* 3CSSP87	AB968639.1 NR_041153.1 NR_115375.1 NR_115366.1	99 99 99 99
SYBC6	1418	*Streptomyces cellostaticus* HUBZM22 *Streptomyces capoamus* JCM 4734 ^a^	HQ853021.1 NR_040856.1	99 99
SYBC7	1399	*Streptomyces cellostaticus* HUBZM22 *Streptomyces cellostaticus* NBRC 12849 ^a^	HQ853021.1 NR_112304.1	99 99
SYBC8	1416	*Streptomyces cinerochromogenes* MC10130 *Streptomyces cinerochromogenes* NBRC 13822 ^a^ *Streptomyces coelescens* CSSP420 *Streptomyces cinerochromogenes* 3CSSP87	AB968639.1 NR_041153.1 NR_115375.1 NR_115366.1	99 99 99 99
SYBC16	1417	*Streptomyces olivogriseus* NBRC 13795 *Streptomyces filipinensis* NBRC 12860 ^a^	AB184486.1 NR_041083.1	99 99
SYBC17	1389	*Streptomyces cellostaticus* HUBZM22 *Streptomyces capoamus* JCM 4734 ^a^	HQ853021.1 NR_040856.1	99 99
SYBC24	1393	*Streptomyces* sp. X4-5 *Streptomyces viridochromogenes* NBRC 13347 ^a^	KT581286.1 NR_112526.1	99 99
SYBC25	1393	*Streptomyces cellostaticus* HUBZM22 *Streptomyces capoamus* JCM 4734 ^a^	HQ853021.1 NR_040856.1	99 99
SYBC26	1393	*Streptomyces indiaensis* LMG 19961 *Streptomyces indiaensis* NBRC 13964 ^a^	AJ781344.1 NR_041155.1	99 99
SYBC27	1422	*Streptomyces cellostaticus* HUBZM22 *Streptomyces cellostaticus* NBRC 12849 ^a^	HQ853021.1 NR_112304.1	99 99

^a^ The best homology 16S rRNA sequences from type material based on a BLAST search of the 16S rRNA sequences among isolates for high similar inference.

**Table 3:** Determination of β-1,3-glucan-degrading activity from culture filtrates of actinomycetes.

Isolates	Total Protein (mg)	Total Activity (U)	Specific Activity (U/mg)
SYBCQL	31.22	3.75	0.12
SYBCA	26.31	23.15	0.88
SYBC6	10.44	5.84	0.56
SYBC7	23.82	13.10	0.55
SYBC8	32.08	3.85	0.12
SYBC16	24.21	10.65	0.44
SYBC17	37.85	38.60	1.02
SYBC24	34.21	6.16	0.18
SYBC25	8.22	2.71	0.33
SYBC26	10.28	4.52	0.44
SYBC27	33.82	4.06	0.12

Note: The volume of culture filtrates is 50 mL.

**Table 4:** The amino acid sequences of β-1,3-glucanase are used to construct the phylogenetic tree. The abbreviations of proteins correspond to positions in the phylogenetic tree of Figure 8.

Organism	Protein Abbreviation	Accession No/PDB No
*Streptomyces sioyaensis*	Curd	AF217415
*Streptomyces matensis* ATCC 23935	LPHase	AB019428
*Streptomyces* sp. S27	BglS27	FJ887899
*Streptomyces clavuligerus* ATCC	SCA1	EFG04651
*Streptomyces acidiscabies*	SCA2	WP_010357589
*Streptomyces coelicoflavus*	SCA55	WP_007387290
*Streptomyces avermitilis*	SCA4	WP_010988837
*Streptomyces avermitilis*	SCA5	WP_010983203
*Streptomyces azureus*	SCA6	GAP51072
*Streptomyces bingchenggensis* BCW-1	SCA7	ADI05411
*Streptomyces canus*	SCA8	WP_020122288
*Streptomyces canus*	SCA9	WP_020117105
*Streptomyces cattleya*	SCA10	WP_014626989
*Streptomyces chartreusis*	SCA11	WP_010043060
*Streptomyces clavuligerus* ATCC 27064	SCA12	EDY52285
*Streptomyces clavuligerus*	SCA13	WP_003957976
*Streptomyces collinus* Tu 365	SCA14	AGS72893
*Streptomyces collinus*	SCA15	WP_020943303
*Streptomyces griseoaurantiacus*	SCA16	WP_006140385
*Streptomyces griseoflavus* Tu4000	SCA17	EFL37893
*Streptomyces griseoflavus*	SCA18	WP_004921557
*Streptomyces griseus*	SCA19	WP_012377737
*Streptomyces himastatinicus*	SCA20	WP_009714916
*Streptomyces hokutonensis*	SCA21	WP_019069886
*Streptomyces hokutonensis*	SCA22	WP_019068505
*Streptomyces hygroscopicus*	SCA23	WP_014676131
*Streptomyces lincolnensis*	SCA24	ANS69291
*Streptomyces malaysiensis*	SCA25	ATL81267
*Streptomyces niveus*	SCA26	WP_023538571
*Streptomyces olivochromogenes*	SCA27	GAX48907
*Streptomyces pratensis*	SCA28	WP_014152186
*Streptomyces prunicolor*	SCA29	WP_019057733
*Streptomyces prunicolor*	SCA30	WP_019059013
*Streptomyces roseochromogenus*	SCA31	WP_023545390
*Streptomyces scopuliridis* RB72	SCA32	PVE09807
*Streptomyces* sp. 351MFTsu5.1	SCA33	WP_020134833
*Streptomyces* sp. 351MFTsu5.1	SCA34	WP_020135556
*Streptomyces* sp. AA4	SCA35	EFL08653
*Streptomyces* sp. ACT-1	SCA36	WP_003964231
*Streptomyces* sp. SPB074	SCA37	WP_008747151
*Streptomyces* sp. SPB78	SCA38	EFL00779
*Streptomyces* sp. SPB78	SCA39	EFK98142
*Streptomyces sparsogenes* DSM 40356	SCA40	OMI34149
*Streptomyces sviceus*	SCA41	WP_007386005
*Streptomyces thermolilacinus*	SCA42	WP_023590036
*Streptomyces violaceusniger*	SCA43	WP_014059092
*Streptomyces viridochromogenes*	SCA44	WP_003994249
*Streptomyces viridosporus* ATCC 14672	SCA45	EFE71345
*Streptomyces viridosporus* ATCC 14672	SCA46	EFE68955
*Streptomyces viridosporus*	SCA47	WP_004986925
*Streptomyces viridosporus*	SCA48	WP_016827665
*Streptomyces viridosporus*	SCA49	WP_016825877
*Streptomyces violaceusniger* Tu 4113	SCA50	AEM85607
*Streptomyces* sp. SCC 2136	SCA51	CAF31374
*Streptomyces zinciresistens*	SCA52	WP_007495688
*Streptomyces zinciresistens*	SCA53	WP_007501949
*Streptomyces coelicoflavus*	SCA54	WP_007389367
*Streptomyces lydicus*	SlgC1SlgC2	CBA11580CBA11566
*Nocardiopsis* sp. F96	BglF	AB244275
*Arthrobacter* sp. Rue61a	Rue	WP_014920770
*Streptomyces* sp. SirexAA-E	SAE	G2NFJ9
*Streptomyces hygroscopicus subsp. jinggangensis* TL01	TL01	AEY93509
*Arthrobacter* sp. NHB-10	GluA2	AB289602
SYBC17	17-W	MH190407
SYBC17	17-Q	MH190408
SYBCQL	*QLK1*	MH190409
*Cellulosimicrobium cellulans* DK-1	DK-1	EU589324
*B. circulans*	GlcA	P23903
*T. maritima* Msb8	*Msb8*	3AZX
*B.circulans* bglM	BglM	AB078775
*Pseudomonas* sp. PE2	GluA1	BAC16331
*Zobellia galactanivorans*	ZgLamA	4BQ1
*Paenibacillus* sp. CCRC 17245	LamA1	ABJ15796
*Corallococcus* sp.	LamC	KX583630
*Mycobacterium fortuitum*	4W65	4W65
*Thermotoga neapolitana*	BglB	Z77856
*Thermotoga petrophila*	TpLam	CP000702
*Pyrococcus furiosus*	pfLamA	2VY0
*Corallococcus* sp.	LamC	KX583630
*Rhodothermus marinus* ITI278	LamR	AAC69707
*Aspergillus fumigatus*	ENGL2	AFUA_2G14360
*Aspergillus fumigatus*	BGT1	AF038596
*Aspergillus fumigatus*	ENGL1	AFUA_1G04260
*Pseudoalteromonas* sp. BB1	ExoP	DQ361032
*Actinosynnema mirum* DSM 43827	DSM	ACU35625
*Micromonospora* sp. L5	MSL5	ADU06434
*Laceyella putida*	LpGluA	LC060791
*Streptomyces coelicolor* A3(2)	CA31	NP_630740
*Streptomyces coelicolor* A3(2)	CA32	NP_625089

**Table 5:** Summary of the purification of recombinant enzymes.

Named	Total Protein (mg)	Total Activity (U)	Specific Activity (U/mg)
QLK1	2.11	138.88	65.82
17-W	1.31	174.09	132.90
17-Q	2.33	34.26	14.70

**Table 6:** Sequences of the target gene primers.

Gene Name	Primer Name	Primer Sequences (5′-3′)	Restriction Site
*17-W*	*17-WF*	GCCGAAGCTTATGGCCTCCCCCCGCCTGCTCC	*Hind* III
	*17-WR*	GCCGTCTAGATCAGCCGACCGTCCACTTCTGGTTGGC	*Xba* I
*17-Q*	*17-QF*	GCCGAAGCTTATGAGTGAAACCTCCGGCATACCCA	*Hind* III
	*17-QR*	GCCGTCTAGATCAGTGACCGAAGTCGAACCAGTTCAC	*Xba* I
*QLK1*	*QLK1-F*	GCCGAAGCTTATGGCTGCTGCCCCACGCACGCGC	*Hind* III
	*QLK1-R*	GCCGTCTAGATCAGCCCAGCGTCCACTTCTGCGCGCC	*Xba* I

## Supplementary Materials

The following are available online. Figure S1: Rarefaction curves based on the OTUs at the cutoff of 97% 16S rRNA sequence similarity, Table S1: Relative abundances (% of total good-quality sequences) of all phyla in each soil sample. The dominant phyla are marked in shade (>1% of good quality sequences in at least one sample), and the total abundances in each soil sample are displayed at the bottom of the latter two lines. Table S2: The abundance of taxa genus levels in soil samples. The taxa represented within the top 30 abundances at the taxa genus levels and beyond the top 30 abundances at the taxa genus levels are classified into other levels.
