# Supplementary material for: Isolation of β-1,3-Glucanase-Producing Microorganisms from Poria cocos Cultivation Soil via Molecular Biology
Source: Molecules. 2018 Jun 27;23(7):1555. doi: 10.3390/molecules23071555 (PMC6100237; doi:10.3390/molecules23071555)
Supplement: Supplementary file 1 [file molecules-23-01555-s001.pdf]

Article

Isolation of β-1,3-Glucanase-Producing Microorganisms from *Poria cocos* Cultivation Soil via Molecular Biology

Qiulan Wu, Xin Dou, Qi Wang, Zhengbing Guan, Yujie Cai and Xiangru Liao *

The Key Laboratory of Industrial Biotechnology, Ministry of Education, School of Biotechnology, Jiangnan University, 1800 Lihu Road, Wuxi, Jiangsu 214122, China; 15861667099@163.com (Q.W.); xin_dou@163.com (X.D.); zhiwen_wei@126.com (Q.W.); 15061886046@163.com (Z.G.); xjj491202524@126.com (Y.C.);

* Correspondence: xrliao@jiangnan.edu.cn; Tel.: +86-1377-1104-596; Fax: +86-0551-8532-7725

Received: 17 May 2018; Accepted: 26 June 2018; Published: date

Supporting information

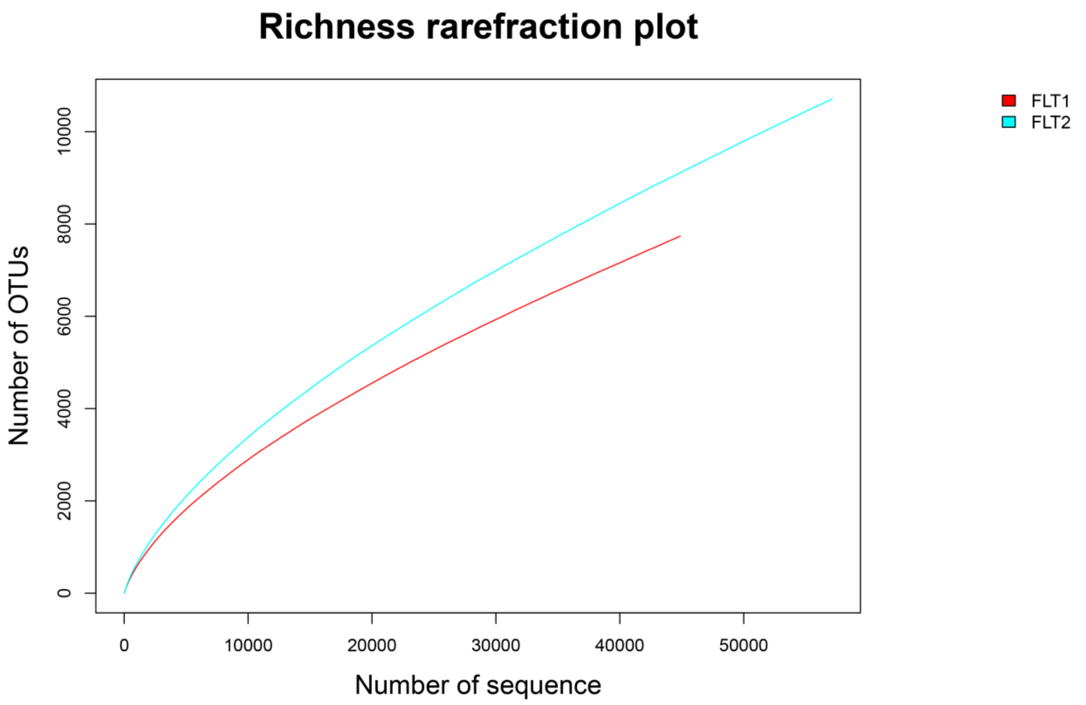

Figure S1. Rarefaction curves based on the OTUs at the cutoff of 97% 16S rRNA sequence similarity.

Table S1. Relative abundances (% of total good-quality sequences) of all phyla in each soil sample. The dominant phyla were marked in shade (>1% of good quality sequences in at least one sample), and the total abundances in each soil sample are displayed at the bottom of the latter two lines.

Phylum	FLT_1 (%)	FLT_2 (%)
Proteobacteria	42.08	47.77
Actinobacteria	18.64	12.60
Acidobacteria	12.41	13.58
Bacteroidetes	5.95	8.00

Verrucomicrobia	4.30	3.80
Firmicutes	3.81	2.63
Gemmatimonadetes	2.80	1.61
Chloroflexi	2.14	1.06
Planctomycetes	2.01	2.27
candidate division WPS-1	1.25	0.33
Candidatus Saccharibacteria	0.74	1.30
Nitrospirae	0.47	0.51
Parcubacteria	0.21	0.79
BRC1	0.16	0.02
Latescibacteria	0.14	0.13
Chlamydiae	0.10	0.46
Armatimonadetes	0.10	0.15
Synergistetes	0.07	0.02
Cyanobacteria	0.06	0.06
Euryarchaeota	0.04	0.01
Fibrobacteres	0.03	0.08
candidate division WPS-2	0.02	0.75
Elusimicrobia	0.02	0.18
Deinococcus-Thermus	0.01	0.03
Aquificae	0.01	0.02
Thaumarchaeota	0.01	0.01
Ignavibacteriae	0.01	0.01
Hydrogenedentes	0.01	0.00
Spirochaetes	0.00	0.15
Crenarchaeota	0.00	0.01
Fusobacteria	0.00	0.00
Thermodesulfobacteria	0.00	0.00
Thermotogae	0.00	0.00
candidate division ZB3	0.00	0.00
**The percentage of dominant phyla**	**96.13**	**94.95**
**The percentage of total identified phyla**	**97.60**	**98.36**

Table S2. The abundance of taxa genus levels in soil sanples.The taxa represented within the top 30 abundance at the taxa genus levels and beyond the top 30 abundence at the taxa genus levels were classified into others.

**FLT_1**		**FLT_2**	
**Genus**	**Score (%)**	**Genus**	**Score (%)**
unclassified	13.90	unclassified	13.90
Sphingomonas	9.56	*Burkholderia*	3.19
Gp4	5.33	Gp1	2.98
Gaiella	4.63	*Sphingomonas*	2.54

Massilia	3.81	Gp2	2.37
Gemmatimonas	2.80	*Mucilaginibacter*	2.34
Glaciimonas	2.50	*Pseudomonas*	2.16
Spartobacteria_genera_incertae_sedis	2.46	*Massilia*	2.13
Gp6	1.93	*Gaiella*	1.88
Streptomyces	1.90	*Phenylobacterium*	1.77
Pseudomonas	1.76	*Bradyrhizobium*	1.68
Burkholderia	1.65	*Gemmatimonas*	1.61
Mucilaginibacter	1.42	*Subdivision3_genera_incertae_sedis*	1.56
WPS-1_genera_incertae_sedis	1.25	Gp3	1.52
Aciditerrimonas	1.23	*Saccharibacteria_genera_incertae_sedis*	1.30
Bacillus	1.12	*Geothrix*	1.28
Novosphingobium	1.11	*Spartobacteria_genera_incertae_sedis*	1.26
Subdivision3_genera_incertae_sedis	1.05	*Labilithrix*	1.25
Gp1	1.01	*Rhizomicrobium*	1.24
Gp16	0.90	*Nevskia*	1.24
Conexibacter	0.87	*Chryseolinea*	1.21
Alkanindiges	0.84	Gp6	1.15
Gp3	0.82	*Kofleria*	1.03
Thermoleophilum	0.82	*Arthrobacter*	0.96
Bradyrhizobium	0.79	*Marmoricola*	0.85
Arthrobacter	0.78	*Candidatus Koribacter*	0.81
Ramlibacter	0.78	*Parcubacteria_genera_incertae_sedis*	0.79
Saccharibacteria_genera_incertae_sedis	0.74	*Aciditerrimonas*	0.77
Phenylobacterium	0.73	*Nocardioides*	0.75
Solirubrobacter	0.69	*WPS-2_genera_incertae_sedis*	0.75
**others**	**30.31**	**others**	**41.53**
**The percentage of total identified genus**	**86.1**	**The percentage of total identified genus**	**86.1**
